# Telemedicina em Cardiologia para Seguimento Ambulatorial de Pacientes com Alto Risco Cardiovascular em Reposta à Pandemia de COVID-19

**DOI:** 10.36660/abc.20200715

**Published:** 2021-01-27

**Authors:** Henrique Turin Moreira, Gustavo Jardim Volpe, Uebe Chade Rezek, Pedro Cunha de Mendonça, Gustavo Corrêa de Almeida Teixeira, Bruno Moreira dos Santos, Anna Paula Gonçalves Olivieri, Ana Julia Abbud Chierice, Henrique Zanqueta Monteiro, Natanael Mendes de Araújo, Benedito Carlos Maciel, Antonio Pazin, André Schmidt

**Affiliations:** 1 Universidade de São Paulo Faculdade de Medicina de Ribeirão Preto Ribeirão PretoSP Brasil Universidade de São Paulo Faculdade de Medicina de Ribeirão Preto , Ribeirão Preto , SP – Brasil

**Keywords:** Betacoronavirus/infecção, COVID-19, Pandemia, Telemedicina, Doença da Artéria Coronariana/complicações, Assistência Ambulatorial

## Introdução

A COVID-19, doença infeciosa causada pelo novo tipo de coronavírus (SARS-Cov-2), apresenta evolução clínica geralmente benigna, embora possa levar à síndrome respiratória aguda grave. Dentre fatores de risco para quadros graves desta doença destacam-se idade avançada e comorbidades como hipertensão, diabetes e outras doenças cardiovasculares. ^1^

Em resposta à epidemia de COVID-19, o volume de atendimentos clínicos eletivos tem sido reduzido. ^2^ Embora pudesse haver expectativa de aumento de eventos cardiovasculares como efeito indesejado dessa reorganização do sistema de saúde, alguns relatos sugerem uma possível redução desses desfechos em países com alta incidência de COVID-19. ^3^ Contudo, fatores associados a essa diminuição ainda não estão bem estabelecidos.

Nesse contexto, a telemedicina tem sido utilizada como método para assistência remota e gerenciamento de consultas médicas, permitindo identificação de pacientes com necessidade de retornos clínicos prioritários, bem como orientações e esclarecimentos à distância aos pacientes. ^4^

O presente estudo avaliou o impacto em curto prazo das medidas de contingência para enfrentamento da pandemia de COVID-19 utilizando-se a telemedicina para seguimento clínico de pacientes de alto risco cardiovascular.

## Métodos

### População do Estudo

Este estudo tranversal avaliou retrospectivamente registros em prontuário médico de teleorientações do serviço de cardiologia do Hospital das Clínicas da Faculdade de Medicina de Ribeirão Preto da Universidade de São Paulo (HC-FMRP-USP), realizadas de 4 a 8 de maio de 2020, dos pacientes que não haviam comparecido em consulta agendada no ambulatório de isquemia miocárdica desde o início da pandemia (reconhecida pela Organização Mundial da Saúde em 11 de março de 2020).

### Teleorientação

A teleorientação é uma das modalidades de telemedicina utilizadas no HC-FMRP-USP para enfrentamento da pandemia de COVID-19, em conformidade com o ofício nº 1756/2020 do Conselho Federal de Medicina e com a portaria do Ministério da Saúde nº 467 de 2020.

No HC-FMRP-USP, a teleorientação médica segue normas institucionais (portaria HCRP 96/2020), podendo ser realizada via contato telefônico, utilizando-se formulário padronizado, com documentação automática no sistema de prontuário eletrônico. O paciente ou seu responsável é sempre informado sobre os motivos do contato telefônico, e questionado sobre a permissão para o registro de dados. Como rotina, houve pelo menos 2 tentativas de contato para cada paciente, realizadas em dias diferentes da semana.

### Dados Clínicos e de Gerenciamento Ambulatorial

Durante a teleorientação, os médicos questionaram ativamente se nos dois meses anteriores ao contato telefônico houve sintomas sugestivos de COVID-19 e realização de teste diagnóstico, além de aparecimento ou piora de dor ou desconforto torácicos, procura por atendimento médico, hospitalização, tratamento recebido, principal motivo pelo não comparecimento ao retorno e necessidade de receitas médicas. Finalmente, o paciente ou seu responsável foi questionado se considerava o reagendamento da consulta melhor ou pior para a saúde do paciente.

### Análise Estatística

Variáveis contínuas são reportadas como média e desvio padrão se distribuídas como uma normal. A normalidade dos dados foi avaliada pelo teste de Shapiro-Wilk. Variáveis categóricas são apresentadas como números absolutos e porcentagens. O nível de significância adotado foi menor que 0,05. O *software* Stata foi utilizado para as análises estatísticas.

### Ética

O presente estudo foi aprovado pelo Comitê de Ética em Pesquisa do HC-FMRP-USP (número do parecer: 4.078.545) e conduzido sob os princípios éticos da declaração de Helsinque e em conformidade com a resolução do Conselho Nacional de Saúde nº 466/2012.

## Resultados

**Figura 1:**
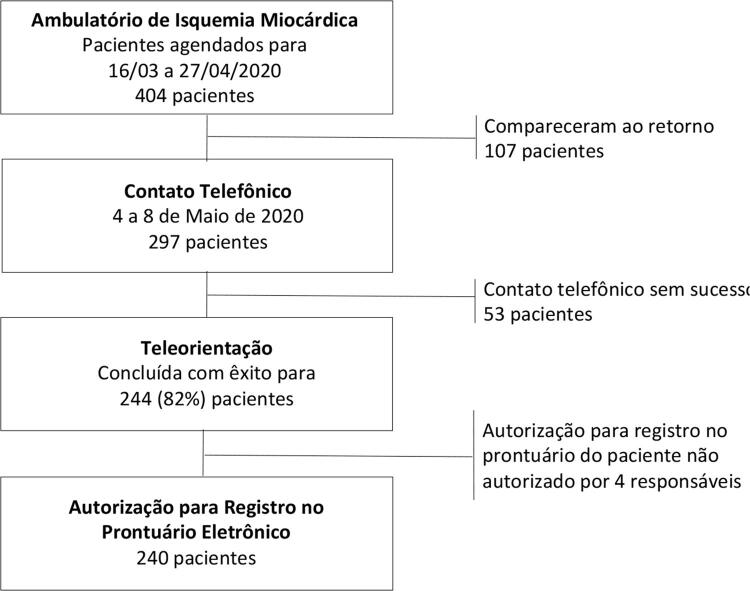
– Seleção dos pacientes incluídos no estudo.

O presente estudo incluiu 240 pacientes selecionados conforme o processo apresentado na Figura 1 . As informações foram fornecidas pelo próprio paciente em 70% dos casos (n=169), enquanto que em 30% dos casos (n=71) foram fornecidas por seu responsável.

A idade média dos pacientes foi de 65±10 anos, 62% homens (n=148) ( Tabela 1 ). Todos os pacientes apresentavam doença arterial coronariana ou isquemia miocárdica, 60% deles com infarto do miocárdio prévio.

**Tabela 1:** – Características clínicas dos 240 pacientes avaliados no estudo

Demográficos	
Idade (anos)	65 ± 10
Sexo masculino	148 (62%)
**Domicílio**	
Estado de São Paulo	235 (98%)
Outros	5 (2%)
**Cidade**	
Ribeirão Preto	68 (28%)
Outras	172 (72%)
**Dados clínicos**	
Hipertensão arterial sistêmica	197 (82%)
Diabetes mellitus	136 (57%)
Tabagismo	
Atual	49 (20%)
Prévio	79 (33%)
**Medicamentos**	
IECA ou BRA	194 (81%)
Estatinas	230 (96%)
**Doença arterial coronariana**	
Com IAM prévio	143 (60%)
Sem IAM prévio	97 (40%)
Intervenção coronariana percutânea	141 (59%)
Cirurgia de revascularização miocárdica	61 (25%)
Fração de ejeção do ventrículo esquerdo*	
Normal	129 (54%)
Intermediária	56 (24%)
Reduzida	54 (23%)

BRA: bloqueador de receptor de angiotensina; IAM = infarto agudo do miocárdio; IECA: inibidor da enzima conversora de angiotensina. *Fração de ejeção do ventrículo esquerdo não foi aferida em 1 participante.

### Evolução Clínica

Sintomas sugestivos de COVID-19 foram reportados por 32 (13%) indivíduos. Coriza e congestão nasal foram os mais frequentes, descritos por 13 pacientes, seguidos por febre (n=10), odinofagia (n=9), piora ou aparecimento de dispneia (n=5) e anosmia (n=2). Não houve hospitalização por COVID-19 ou realização de teste para SARS-CoV-2.

**Figura 2:**
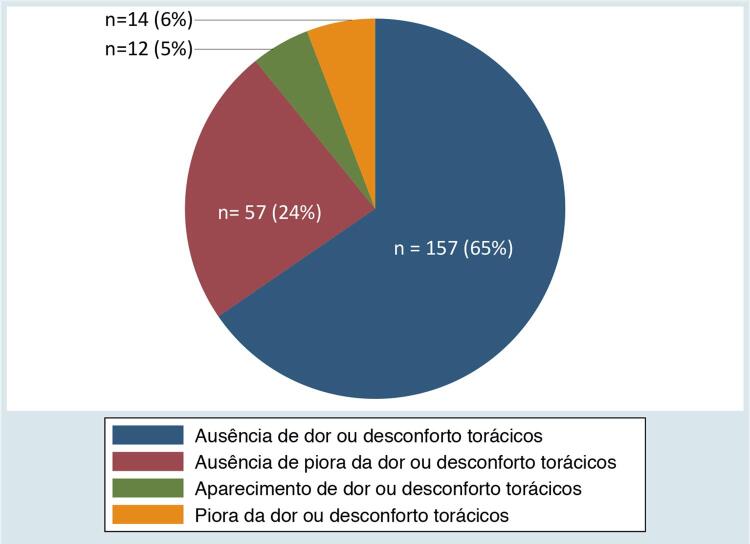
– Presença de sinais de alarme durante seguimento ambulatorial de pacientes com doença arterial coronariana crônica.

Aparecimento ou piora de dor ou desconforto torácico foram relatados por 12 (5%) e 14 (6%) pacientes, respectivamente. Desses 26 pacientes, 13 procuraram atendimento de urgência, sendo que 3 deles foram internados, 1 destes em unidade de terapia intensiva com diagnóstico de infarto agudo do miocárdio (IAM) tratado com intervenção coronariana percutânea (ICP) em outro serviço ( Figura 2 ). Os outros dois pacientes hospitalizados não foram admitidos em unidade de terapia intensiva; um deles relatou síndrome coronariana aguda e ICP em outro serviço, enquanto o outro referiu não saber o diagnóstico da internação. Houve relato de óbito de uma paciente do sexo feminino, de 80 anos, com fração de ejeção do ventrículo esquerdo reduzida, porém não tivemos acesso ao atestado de óbito.

### Seguimento Ambulatorial

**Figura 3:**
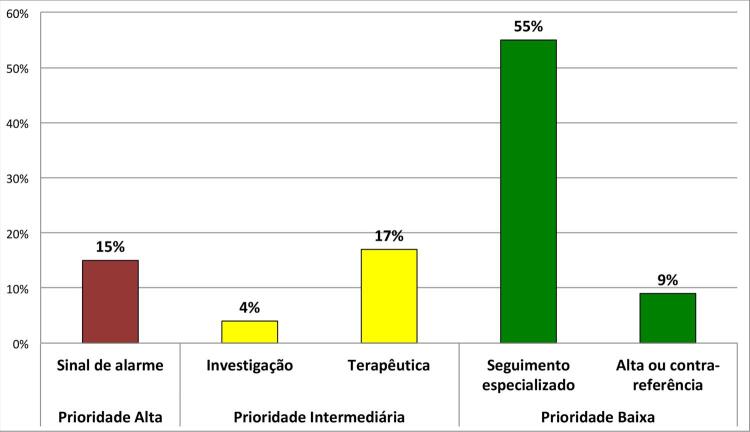
– Tipos de retornos triados através de teleorientações.

A maioria dos pacientes (80%) não compareceu à consulta, referindo ter seguido recomendações do HC-FMRP-USP, enquanto 13% dos pacientes relatou não comparecimento devido ao medo de contaminação pelo SARS-CoV-2 nas dependências do hospital e 3% dos pacientes não obteve meio de transporte para a consulta, sendo que 4% dos pacientes relatou outros motivos (tabela suplementar). Retornos “prioridade alta”, “intermediária” e “baixa” foram agendados para 15%, 22% e 63% dos pacientes, respectivamente ( Figura 3 ).

Necessidade de receita médica foi apontada por 8% dos pacientes. Metade dos pacientes considerou que o reagendamento da consulta foi melhor para a sua saúde, enquanto que essa medida foi considerada indiferente ou pior para a própria saúde por 30% e 20% dos pacientes, respectivamente.

## Discussão

O presente estudo avaliou o impacto em curto prazo de estratégia para seguimento clínico de pacientes de alto risco cardiovascular com o uso de telemedicina em resposta à pandemia de COVID-19. Dentre os principais achados, destaca-se que 11% dos pacientes avaliados apresentou agravamento do quadro cardiovascular nos primeiros meses da pandemia e apenas metade desses procurou atendimento médico por esse motivo. Além disso, uma importante parcela dos pacientes relatou receio em comparecer ao serviço de saúde devido ao potencial risco de contaminação intra-hospitalar pelo SARS-Cov-2. Nesse cenário, a aplicação de teleorientação mostrou-se bastante exequível, de boa aceitação pelos pacientes e bastante útil no planejamento dos retornos clínicos baseados em categorias de prioridade.

Desde os primeiros relatos de casos de COVID-19, há relatos de redução do número de atendimentos por condições cardiovasculares agudas graves. ^5^ Dados de laboratórios de cateterismo dos EUA mostraram redução de 38% no número de ICP para IAM durante a fase inicial da epidemia de COVID-19 no país. ^3^ Inquérito mais recente envolvendo 141 países indica que em cerca de dois terços deles houve redução maior que 40% do número de admissões hospitalares por IAM nos primeiros meses da pandemia. ^6^

Dentre as hipóteses levantadas para esses achados, a mais frequentemente apresentada tem sido o medo dos pacientes de se contaminar pelo SARS-Cov-2 nos ambientes hospitalares, como apontado em recente relato de caso no Brasil. ^7^ No presente estudo, 13% referiram que o principal motivo para o não comparecimento foi o medo de contaminação intra-hospitalar.

Este estudo contribui para o avanço do conhecimento no campo da telecardiologia, mostrando elevada exequibilidade do processo, com grande aceitação dos pacientes seguidos na instituição. A ação implementada proporcionou ferramenta eficaz de gerenciamento dos retornos clínicos, marcados como prioritários para 15% dos pacientes contactados, enquanto os outros 85% puderem manter o distanciamento social preconizado durante essa pandemia. Além disso, outras necessidades puderam ser contempladas, como a identificação da necessidade de receitas médicas, levantadas em 8% dos casos.

A teleorientação não foi concluída com êxito em 18% dos casos, não sendo possível descartar que a proporção de pacientes que tenha apresentado piora clínica, e até mesmo óbito, seja ainda maior do que a observada.

## Conclusões

A telemedicina em cardiologia para o enfrentamento da COVID-19 é altamente exequível, bastante eficaz e de grande aceitação pelos pacientes, permitindo triagem de casos prioritários e gerenciamento dos retornos ambulatoriais.
