# Supplementary material for: Telemedicina em Cardiologia para Seguimento Ambulatorial de Pacientes com Alto Risco Cardiovascular em Reposta à Pandemia de COVID-19
Source: Arq Bras Cardiol. 2021 Jan 27;116(1):153–7. [Article in Portuguese] doi: 10.36660/abc.20200715 (PMC8159502; doi:10.36660/abc.20200715)
Supplement: Translation

# Translation: Telemedicine in Cardiology for Outpatient Follow-Up of Patients at High Cardiovascular Risk in Response to the COVID-19 Pandemic

**Authors:** Henrique Turin Moreira, Gustavo Jardim Volpe, Uebe Chade Rezek, Pedro Cunha de Mendonça, Gustavo Corrêa de Almeida Teixeira, Bruno Moreira dos Santos, Anna Paula Gonçalves Olivieri, Ana Julia Abbud Chierice, Henrique Zanqueta Monteiro, Natanael Mendes de Araújo, Benedito Carlos Maciel, Antonio Pazin, André Schmidt

**Affiliations:** 1 Brazil Universidade de São Paulo Faculdade de Medicina de Ribeirão Preto, Ribeirão Preto, SP – Brazil

**Keywords:** Betacoronavirus/infection, COVID-19, Pandemics, Telemedicine, Coronary Artery Disease/complications, Ambulatory Care

## Introduction

COVID-19, an infectious disease caused by the new type of coronavirus (SARS-Cov-2), usually shows a benign clinical course, although it can lead to acute respiratory distress syndrome. The main risk factors for the severe form of COVID-19 include older age and presence of comorbidities, such as diabetes, hypertension and other cardiovascular diseases.^1^

In response to the COVID-19 pandemic, elective medical appointments have been reduced.^2^Even though an increase in cardiovascular events as an adverse effect of this healthcare system reorganization would be expected, some reports have suggested a possible reduction of such outcomes in countries with high prevalence of SARS-CoV-2 infection.^3^However, the mechanisms related to this decline are not well understood.

In this context, telemedicine has been used as a strategy for remote assistance and management of patient care, hence allowing for the identification of those in need of a priority medical appointment, as well as remote guidance.^4^

Therefore, this study aimed at assessing the short-term results of measures adopted in response to COVID-19 pandemic by using telemedicine in the following-up of patients at high cardiovascular risk.

## Methods

### Study Population

This cross-sectional study retrospectively assessed data from patient medical records of teleorientation services performed by cardiologists in the Hospital das Clínicas of the Medical School of Ribeirão Preto, University of São Paulo (HCFMRP-USP), between May 4 and 8, 2020, of patients treated in the ischemic heart disease outpatient clinic who had not attended a scheduled medical appointment since the COVID-19 outbreak, which was declared a pandemic by the World Health Organization (WHO) on March 11, 2020.

### Teleorientation

Teleorientation is a modality of telemedicine adopted by the HCFMRP-USP as a strategy during the COVID-19 pandemic, in accordance with the Brazilian Federal Council of Medicine (CFM) Official Letter No. 1,756/2020 and the Ordinance No. 467 of the Ministry of Health (MoH), issued on March 20, 2020.

At the HCFMRP-USP, the use of teleorientation follows institutional rules (HCFMRP-USP Ordinance 96/2020), and can be performed by telephone call, using a standardized questionnaire from the institutional electronic medical record system. The patient (or a representative person) is always informed about the reasons for the contact and asked about consent to be recorded. As a routine if necessary, at least two telephone call attempts were made in different days.

### Clinical Data and Management of Outpatient Care

During teleorientation, physicians actively asked whether in the last two weeks from the phone call the patient had any symptoms suggestive of COVID-19 and whether the patient was subjected to laboratory test for SARS-CoV-2. Moreover, the patient was asked about emergence or worsening of chest pain or discomfort, seeking for emergency room, need of hospitalization, treatments received, main reason for not showing up in the outpatient clinic return appointment, and need for renewing medical prescriptions. Finally, the patient or a representative was asked whether the consultation rescheduling had been better or worse for the patient’s health.

### Statistical analysis

Continuous variables are reported as mean and standard deviation, if normally distributed. Data normality was assessed by the Shapiro-Wilk test. Categorical variables are presented as absolute numbers and percentages. The significance level adopted was lower than 0.05. STATA software was used to perform statistical analysis.

### Ethics

This study was approved by the local HCFMRP-USP Research Ethics Committee (protocol n^o^4.078.545), conducted under the ethical principles of the Declaration of Helsinki, and developed in accordance with the Resolution no. 466/2012 of the National Health Council.

## Results

The study included 240 patients, as shown in the flowchart of patient enrollment process ( Figure 1 ). Data were provided by the patient in 70% of the cases (n = 169), whereas in 30% of the cases (n = 71) data were provided by a patient representative.

**Figure 1:** – Patient enrollment.

Patients mean age was 65 ± 10 years, 62% men (n = 148) ( Table 1 ). All patients had coronary artery disease or myocardial ischemia, 60% of them had prior myocardial infarction.

**Table 1:** – Clinical characteristics of the 240 patients assessed in the study

Demographic	
Age (years)	65 ± 10
Men	148 (62%)
**State of residence**	
State of Sao Paulo	235 (98%)
Others	5 (2%)
**City**	
Ribeirao Preto	68 (28%)
Other	172 (72%)
**Clinical data**	
Hypertension	197 (82%)
Diabetes	136 (57%)
Smoking	
Current	49 (20%)
Former	79 (33%)
**Medication in use**	
ACE or ARB	194 (81%)
Statins	230 (96%)
**Coronary artery disease**	
With Previous myocardial infarction	143 (60%)
No previous myocardial infarction	97 (40%)
Percutaneous coronary intervention	141 (59%)
Coronary artery bypass graft	61 (25%)
Left ventricular ejection fraction*	
Normal	129 (54%)
Mid-range	56 (24%)
Reduced	54 (23%)

ACE: angiotensin converting enzyme; ARB: angiotensin receptor blocker. *Left ventricular ejection fraction was not assessed in one patient. References

### Clinical Course

Symptoms suggestive of COVID-19 were reported by 32 (13%) patients. Rhinorrhea and nasal congestion were the most frequent symptoms, described by 13 individuals, followed by fever (n = 10), odynophagia (n = 9), worsening or onset of dyspnea (n = 5), and anosmia (n = 2). No patient reported hospitalization by COVID-19 or testing for SARS-CoV-2 infection.

New onset or worsening chest pain was reported by 14 (6%) and 12 (5%) patients, respectively. Of these 26 patients, 13 individuals were admitted to emergency rooms, and 3 of them were hospitalized, 1 due to myocardial infarction and 1 due to acute coronary syndrome. Both patients were treated with percutaneous coronary intervention (PCI). A third patient was unable to report the diagnosis that led to the hospitalization. One death was reported: a woman aged 80 years, with reduced left ventricular ejection fraction. Unfortunately, we did not have access to the death certificate to assert the cause of death.

### Outpatient Follow-up

The majority of patients (80%) rescheduled the medical appointment, following the recommendations of the HCFMRP-USP, while 13% of patients reported non-attendance due to fear of nosocomial infection with SARS-Cov-2, 3% of the patients had no means of transport to get to the appointment, and 4% of the patients reported other reasons (supplementary table). High, intermediate and low priority medical appointments were scheduled for 15%, 22% and 63% of patients, respectively.

The need for renewal of prescriptions was reported by 8% of the patients. Half of the patients contacted considered that the rescheduling was better for their health, while this strategy was considered neutral or worse by 30% and 20% of the patients, respectively.

## Discussion

This study assessed the short-term results of strategies to the following-up of outpatients at high cardiovascular risk by means of telemedicine in response to the COVID-19 pandemic. As the main findings, 11% of the contacted patients had worsening of their cardiovascular condition in the first months of the pandemic, but only half of those patients sought medical evaluation for that reason. Moreover, an important proportion of patients reported fear of attending health facilities due to the potential risk of in-hospital contamination by SARS-CoV-2. In this scenario, teleorientation was highly feasible, of good acceptance by patients and very useful in the management of medical appointments based on clinical priorities.

Since the first cases of COVID-19, some investigations have reported a decrease in the medical care demand due to cardiovascular events.^5^Data from catheterization laboratories from the US have shown an estimated 38% reduction in emergency ST-segment-elevation myocardial infarction (STEMI) activations at the beginning of the pandemic breakout in that country.^3^Similarly, a more recent study involving 141 countries has indicated that in about two thirds of them there was a decrease of 40% or more in hospital admissions due to STEMI during the first months of the pandemic.^6^

A frequent hypothesis to those findings has been fear of SARS-CoV-2 infection in medical facilities, as has been demonstrated recently in a Brazilian case report.^7^In this study, 13% of patients reported fear of in-hospital infection as the main reason for not attending the previously scheduled medical appointment.

In addition, this study contributes to advance the current knowledge of the telemedicine field, by showing its high feasibility and good acceptance by the patients. The telemedicine-based strategy used in this study allowed for efficient management of medical appointments, scheduled as priority for 15% of contacted patients, while other 85% of patients could postpone their medical visit and hence remain in social distancing. In addition, other needs could be fulfilled, such as medical prescription renewal, which was required for 8% of the contacted patients.

Teleorientation was not successfully completed in 18% of the cases, thus it is not possible to rule out that the proportion of patients who had clinical worsening, and even death rate, was greater than the observed.

## Conclusions

Telemedicine in cardiology in response to the COVID-19 pandemic was highly feasible, very effective and widely accepted by patients, allowing for the screening of priority cases and the management of outpatient return appointments.

**Figure 2:** – Presence of warning symptoms reported by outpatients with chronic coronary artery disease.

**Figure 3:** – Medical visits screened by teleorientation.
